# Is allergy to peanuts and nuts a predictive factor for asthma development?

**DOI:** 10.1186/2045-7022-5-S3-P168

**Published:** 2015-03-30

**Authors:** Mirjana Zivanovic, Marina Atanaskovic-Markovic

**Affiliations:** 1 Special Hospital for Lung Diseases, Sokobanja, Serbia; 2 Faculty of Medicine, University of Belgrade, University Children's Hospital, Belgrade, Serbia

## Introduction

Food allergy is an immunologically mediated adverse reaction to food. Peanuts and nuts are common cause of food allergy in both children and adults, and clinical picture may be serious and life threatening. This type of food allergy persists for entire life, while cross-reactivity is not uncommon due to homology among most nut allergens.

## Aim

The aim of this paper was to determine the significance of peanut and nuts allergy in asthma development.

## Material and methods

The aim of the research was analyzed on a sample of 208 children aged between 6 months and 6 years who were evaluated to food allergens. Each child underwent skin prick test, prick to prick test and specific IgE testing.

## Results

The total sample included 105 boys and 103 girls. Peanut allergy was present in 37 (17.79%) and nuts allergy in 32 (15.38%) of children. Positive family history to atopy was present in 52% and positive personal history in 54% of cases. Asthma developed in 30 (81.08%) children allergic to peanuts and 24 (75%) children allergic to nuts.

## Conclusion

Peanut allergy is a predictive factor for asthma development; however, the effects of positive personal and family history must not be underestimated.

